# High-frequency repetitive transcranial magnetic stimulation improves functional recovery by inhibiting neurotoxic polarization of astrocytes in ischemic rats

**DOI:** 10.1186/s12974-020-01747-y

**Published:** 2020-05-06

**Authors:** Ye Hong, Qian Liu, Mengna Peng, Maosheng Bai, Juanji Li, Rui Sun, Hongquan Guo, Pengfei Xu, Yi Xie, Yunzi Li, Ling Liu, Juan Du, Xinfeng Liu, Bin Yang, Gelin Xu

**Affiliations:** 1 Department of Neurology, Jingling Hospital, Nanjing University School of Medicine, 305# East Zhongshan Road, Nanjing, 210002 Jiangsu China; 2 Department of Orthopedics, Nanjing Tongren Hospital, Nanjing, 210002 Jiangsu China; 3 Department of Orthopedics, Nanjing Hospital of Chinese Medicine Affiliated to Nanjing University of Chinese Medicine, Nanjing, 210002 Jiangsu China; 4 Department of Neurology, Jinling Hospital, Nanjing Medical University, Nanjing, 210002 Jiangsu China; 5 Department of Neurology, Changhai Hospital, Second Military Medical University/Naval Medical University, Shanghai, 210000 China; 6 Department of Neurology, Jinling Hospital, Southern Medical University, Nanjing, 210002 Jiangsu China; 7 Stroke Center & Department of Neurology, The First Affiliated Hospital of USTC, Division of Life Sciences and Medicine, University of Science and Technology of China, Hefei, 230036 Anhui China; 8 Department of Ultrasonography, Jingling Hospital, Nanjing University School of Medicine, 305# East Zhongshan Road, Nanjing, 210002 Jiangsu China

**Keywords:** Ischemic stroke, Astrocytic polarization, rTMS

## Abstract

**Background:**

Repetitive transcranial magnetic stimulation (rTMS) is a noninvasive treatment for ischemic stroke. Astrocytes regulation has been suggested as one mechanism for rTMS effectiveness. But how rTMS regulates astrocytes remains largely undetermined. There were neurotoxic and neuroprotective phenotypes of astrocytes (also denoted as classically and alternatively activated astrocytes or A1 and A2 astrocytes) pertaining to pro- or anti-inflammatory gene expression. Pro-inflammatory or neurotoxic polarized astrocytes were induced during cerebral ischemic stroke. The present study aimed to investigate the effects of rTMS on astrocytic polarization during cerebral ischemic/reperfusion injury.

**Methods:**

Three rTMS protocols were applied to primary astrocytes under normal and oxygen-glucose deprivation/reoxygenation (OGD/R) conditions. Cell survival, proliferation, and phenotypic changes were assessed after 2-day treatment. Astrocytes culture medium (ACM) from control, OGD/R, and OGD/R + rTMS groups were mixed with neuronal medium to culture neurons for 48 h and 7 days, in order to explore the influence on neuronal survival and synaptic plasticity. In vivo, rats were subjected to middle cerebral artery occlusion (MCAO), and received posterior orbital intravenous injection of ACM collected from different groups at reperfusion, and at 3 days post reperfusion. The apoptosis in the ischemic penumbra, infarct volumes, and the modified Neurological Severity Score (mNSS) were evaluated at 1 week after reperfusion, and cognitive functions were evaluated using the Morris Water Maze (MWM) tests. Finally, the 10 Hz rTMS was directly applied to MCAO rats to verify the rTMS effects on astrocytic polarization.

**Results:**

Among these three frequencies, the 10 Hz protocol exerted the greatest potential to modulate astrocytic polarization after OGD/R injury. Classically activated and A1 markers were significantly inhibited by rTMS treatment. In OGD/R model, the concentration of pro-inflammatory mediator TNF-α decreased from 57.7 to 23.0 рg/mL, while anti-inflammatory mediator IL-10 increased from 99.0 to 555.1 рg/mL in the ACM after rTMS treatment. The ACM collected from rTMS-treated astrocytes significantly alleviated neuronal apoptosis induced by OGD/R injury, and promoted neuronal plasticity. In MCAO rat model, the ACM collected from rTMS treatment decreased neuronal apoptosis and infarct volumes, and improved cognitive functions. The neurotoxic astrocytes were simultaneously inhibited after rTMS treatment.

**Conclusion:**

Inhibition of neurotoxic astrocytic polarization is a potential mechanism for the effectiveness of high-frequency rTMS in cerebral ischemic stroke.

## Introduction

Clinical studies have confirmed that repetitive transcranial magnetic stimulation (rTMS) is effective for treating stroke patients with dysphagia [1], aphasia [2, 3], motor dysfunctions, and chronic pain [4, 5]. Poststroke improvement could be long-lasting after rTMS treatment. As reported, dysphagia improvement can last for 3 weeks, and aphasia improvement can persist for up to 8 months after the last treatment of rTMS [1, 2]. Despite the wide application of rTMS in the clinical setting, the underlying mechanisms remain largely undetermined [6]. Previous studies about rTMS mechanisms have mainly focused on influences on neurons. It has been well-accepted that high-frequency stimulation (≥ 5 Hz) improves neuron excitatory at the lesioned cortical areas, while low-frequency stimulation (≤ 1 Hz) inhibits neuron excitatory at the contralateral brain to reduce interhemispheric inhibition and facilitate neuronal activity in the compensation regions [7, 8]. In addition, researches that used the ischemia-reperfusion model and prolonged ischemic model revealed that rTMS could also protect neurons against death, and alter blood flow and metabolism in the brain [9–11]. However, there is a lack of data on the effect of rTMS on non-neural cells, such as astrocytes.

Studies conducted on the primary astrocytes and other disease models over the past two decades have made it clear that astrocytes are the cellular effectors of rTMS [12, 13]. Astrocytes possess ion channels and neurotransmitter receptors, and play an important role in synaptic transmission. Hence, it is possible that rTMS could change the astrocyte cell membrane potential and cell function [14]. In vitro, high-frequency rTMS (50 Hz) could boost Ca^2+^ inflow, increase interleukin-6 release, and promote astrocyte proliferation [15, 16]. The glial fibrillary acid protein (GFAP) levels in the astrocytes of gerbils and rats exposed to rTMS were increased following rTMS stimulation [17, 18]. Additionally, rTMS stimulation increased the migratory capacity of reactive white matter astrocytes to a CNS lesion in an animal model of spinal cord injury [19]. Both glial activation and associated neuroinflammation could be attenuated by high-frequency rTMS at the site of focal brain injury and spinal cord injury in rats [20, 21]. These results indicated that rTMS may reduce neuroinflammation by influencing astrocytic activation.

Astrocytes are a dominant non-neuronal cell population, and therapies aimed at modulating astrocytes in stroke patients are associated with improved outcomes [22]. Upon injury, astrocytes were activated, and these have been reported to play dual roles, depending on phenotype [23]. Classically activated astrocytes exert neurotoxic effects by releasing pro-inflammatory mediators, while alternatively astrocytes perform neuroprotective effects by secreting anti-inflammatory mediators. The recently identified A1 and A2 astrocytes had the same functions as classically and alternatively activated astrocytes [24]. Inducible nitric oxide synthase (iNOS) and arginase 1 (Arg1) have been respectively regarded as markers of classically and alternatively activated astrocytes [25]. Furthermore, compliment C3 (C3) and S100 calcium binding protein A10 (S100A10) have been respectively regarded as markers of A1 and A2 astrocytes [24]. In the present study, iNOS and C3 were combined to mark neurotoxic astrocytes and Arg1 and S100A10 were combined to mark neuroprotective astrocytes. Previous studies have reported both types of astrocytes in cerebral ischemic models. Reactive astrocytes exhibited an enrichment of transcripts associated with A2 phenotype at 72 h after transient middle cerebral artery occlusion (tMCAO) [26]. However, a large number of neurotoxic genes were induced at the same time in astrocytes. In primary astrocytes exposed to oxygen-glucose deprivation and reoxygenation (OGD/R) injury, classically activated markers were highly induced at 24 h and 48 h post-reoxygenation [25]. Furthermore, targeting A1 astrocytic polarization is a potential strategy in developing new drugs for treating Alzheimer’s disease [27]. Hence, the phenotypic transition of astrocytes after ischemic stroke might also be a therapeutic target to control neuroinflammation and promote functional recovery.

We hypothesized that rTMS could modulate astrocytes polarization in cerebral ischemic models to promote functional recovery. In order to test this hypothesis, rTMS at frequency of 1, 5, or 10 Hz was delivered to normal astrocytes or astrocytes exposed to OGD/R injury. Then, the cell survival, proliferation, and polarization status were investigated. The 10 Hz rTMS stimulation could inhibit neurotoxic polarization and promote the neuroprotective polarization of astrocytes both in vitro and in vivo. The effects of rTMS-modulated astrocytes on neural survival, neurite plasticity, and neurological functional recovery were systematical assessed. To our knowledge, this is the first study to investigate the impact of rTMS on astrocytic polarization after cerebral ischemia.

## Methods

### Animals

Male adult Sprague-Dawley rats weighing 250–270 g, female pregnant rats of 16–18 days, and puppies within 24 h of birth were purchased from Model Animal Research Institute of the Nanjing University (Nanjing, Jiangsu, China). Animals were housed under a 12-h light/dark cycle at approximately 25 °C and a relative humidity of 65% and provided ad libitum access to food and water. Experimental protocols were approved by the Animal Subject Review Board of Jinling Hospital and were conducted in accordance with recommendations from the Guide for the National Institutes of Health for the Care and Use of Laboratory Animals (NIH Publications No. 8023, revised 2011). The utmost efforts were made to minimize the number of animals used and their suffering.

### Primary astrocytes and neuron culture

Primary cortical neurons and primary astrocytes were cultured and purified as previously reported [23]. Primary neurons were dissected from the cortex of embryonic SD rats (E16-18), while astrocytes were isolated from neonatal puppies within 24 h of birth. Cortical tissues were digested with 0.125% trypsin (Gibco, Rockville, MD, USA), homogenized by pipetting, and filtered with a 100-μm cell strainer (Biologix, Shandong, China). The cells were then centrifuged and re-suspended in Dulbecco’s modified Eagle’s medium (DMEM; Hyclone, Logan, UT, USA) with 10% fetal bovine serum (FBS; Gibco) and penicillin-streptomycin (Gibco). For neurons, suspension cells were seeded in poly-d-lysine (PDL; Sigma-Aldrich, St. Louis, Missouri, USA) coated coverslips and replaced with neurobasal medium containing 2% B27 and 1% glutamax (Thermo Fisher Scientific, MA, USA) 2 h later. For astrocytes, the suspension cells were seeded in flasks (Costar, NY, USA) coated with PDL to obtain mixed glial cells. Then, 10–14 days later, the flasks were shaken at 200 rpm for 4–6 h at 37 °C. Adherent cells were re-seeded in cell culture dishes to obtain astrocytes.

### Oxygen-glucose deprivation and reoxygenation

OGD was conducted according to a previously established protocol [28]. Briefly, cells seeded in the culture dishes were incubated with glucose-free DMEM medium in an anaerobic chamber equipped with AnaeroPack-Anaero (MGC, Japan) at 37 °C. After 6 h, cells were returned to normal incubator and incubated with the initial culture medium for reoxygenation.

### rTMS

A customized magnetic stimulator (MagPro X 100 with Magoption, Tonica, DK) with a C-100 circular coil (20 mm in inner diameter with 1.9 T peak magnetic stimulator output) was used to stimulate primary cells and middle cerebral artery occlusion (MCAO) rats. The stimulation intensity was set at 120% of the average resting motor threshold (RMT) of rats. The detection method of RMT was based on a previous study [29]. In our study, the RMT was 30% of the maximum stimulator output.

**Fig. 1:**
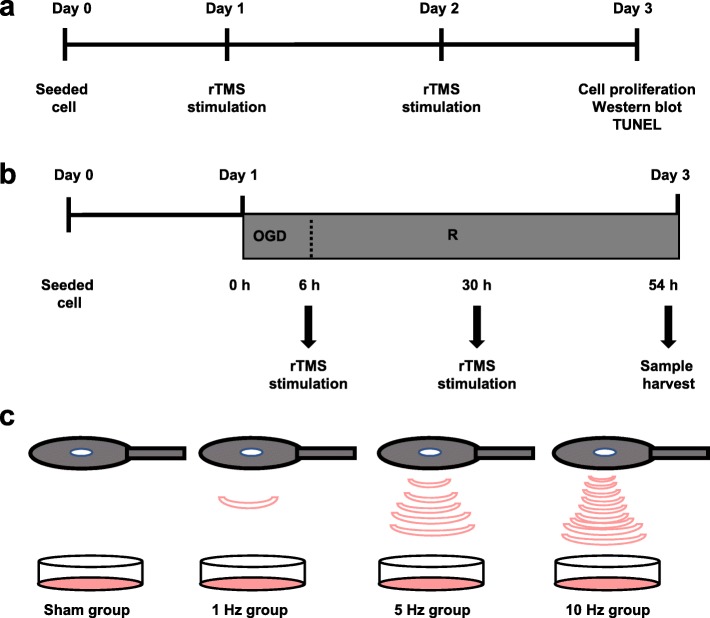
The experiment timelines indicating when each procedure was performed. **a** The rTMS stimulation for normal astrocytes. On day 0, equal number of astrocytes were seeded in growth medium. On day 1 and day 2, cells received rTMS stimulation for 10 min. After the stimulation, the cells were incubated in the cell incubator. At 24 h post the last stimulation, cells were harvested for indicated experiments. **b** rTMS stimulation for astrocytes exposed to OGD/R injury. Twenty-four hours after the seeding of astrocytes, the culture medium was changed into deoxygenated, glucose-free balanced salt solution and placed into a hypoxia chamber for 6 h. After this OGD injury, astrocytes were cultured with normal culture and received rTMS for 10 min. The second rTMS was performed at 24 h after reperfusion. Samples were collected at 24 h after the second stimulation. **c** A scheme of rTMS treatment in primary astrocytes. The cultured cells were divided into the sham group, the 1 Hz group, the 5 Hz group, and the 10 Hz group. rTMS, repetitive transcranial magnetic stimulation. *OGD* oxygen and glucose deprivation, *R* reoxygenation, *TUNEL* terminal of dUTP nick end-labeling

In vitro, the magnetic coil was placed and positioned 1 cm away from the culture dish. Primary astrocytes in normal condition or exposed to OGD/R injury were divided into four groups, as sham group (placing the culture dishes without magnetic stimulation), the 1 Hz group, the 5 Hz group, and the 10 Hz group to deliver 600 pulses in 10 min every day for two consecutive days. An overview of the experiment design is shown in Fig. 1.

In vivo, conscious rats were treated with 10 Hz rTMS for 10 min per day. The treatment started at 24 h after the ischemia-reperfusion and lasted for 7 days. The stimulation site was located above the ipsilateral primary motor cortex (right M1 region) as determined by a stereotactic apparatus (around 5 mm to the right of bregma). Most procedures were based on previous studies [30, 31].

### LPS treatment

LPS from *Escherichia coli* 0111: B4 (prepared by phenolic extraction and gel filtration chromatography) was obtained from Sigma-Aldrich (St. Louis, MO). After OGD, primary astrocytes were cultured with normal medium containing LPS (100 ng/mL). Same volume of PBS was used as control treatment. Then, these cells were applied for rTMS experiments. Eight hours later, cell cultures were replaced with normal culture medium without LPS or PBS. Astrocyte-conditioned media were collected at 48 h post-OGD.

### Transient middle cerebral artery occlusion

The rats were anesthetized with 2–3% isoflurane (RWD Life Science, Shenzhen, China). The MCAO surgery was operated according to a previous study [15]. A silicon-coated nylon monofilament was inserted into the right middle cerebral artery until mild resistance was felt. Blood flow reduced more than 70% of that at the baseline, as monitored by a Laser Doppler flowmeter (LDF; Perimed PF5000, Stockholm, Sweden), was deemed as successful occlusion. After 90 min of occlusion, the monofilament was withdrawn for reperfusion. During the surgical procedures, body temperature was maintained at 37 ± 0.5 °C using a heat lamp. In the sham group, rats underwent the same procedures except that the middle cerebral artery was not occluded after the neck incision.

### Astrocyte-conditioned media collection

To obtain astrocytes-conditioned media (ACM), primary astrocytes were seeded at 3 × 10^6^ cells/dish in 6-cm cell culture dishes. After treating with OGD for 6 h, cells were washed with PBS and cultured in fresh culture media followed by rTMS stimulation. Conditioned astrocytes media were collected at 48 h post-OGD and centrifuged at 1000 rpm for 5 min to remove cellular debris. Then, the ACM were applied to ELISA experiment or mixed with primary neuronal cell culture (1:1) to detect the ACM effects on neuronal apoptosis and plasticity.

For posterior orbital vein injection, ACM was concentrated using 10 kDa-membrane centrifuge tubes (Millipore, UFC901024) and spun for a total of 30 min at 4000 g at 21 °C (about 12× final volume). Single aliquots of concentrated ACM were frozen at − 80 °C until use.

### ACM therapy

Under anesthesia, each rat received posterior orbital intravenous injection of 160 μL concentrated ACM at the time of MCAO reperfusion, and at 3 days post reperfusion.

### ELISA

The concentrations of tumor necrosis factor alpha (TNF-α) and IL-10 in all ACM samples were measured by the specific ELISA kit (Neobioscience, China). These measurements were based on the instructions of manufacturer.

### Immunofluorescence and TUNEL staining

Samples were fixed with 4% paraformaldehyde for 30 min, and permeabilized with 0.1% Triton X-100 for 10 min, then blocked with 5% BSA in PBS for 60 min at 37 °C and incubated with the indicated primary antibodies at 4 °C overnight. Primary antibodies against GFAP, S100A10, and iNOS were purchased from Abcam (Cambridge, UK). C3 antibody was from Santa Cruz Biotechnology (CA, USA). Arg1 antibody was bought from Cell Signaling Technology (MA, USA). After four washes with PBS, the samples were incubated with appropriate secondary antibodies (1:200) and DAPI (Sigma-Aldrich, USA). Finally, observing under a FluoView FV10i confocal laser scanning microscope (Olympus, Japan) or an Olympus BX51 microscope.

Apoptotic neurons were immunostained with NeuN (1:200, Abcam, UK) and terminal deoxynucleotidyl transferase-mediated dUTP nick-end labeling (TUNEL; Beyotime, Nanjing, China) according to manufacturer’s protocol. Positively labeled cells were calculated by ImageJ software.

### Western blot analysis

Total proteins from brain tissues or cultured cells were lysed in RIPA buffer (Cell Signaling Technology, MA, USA) with 1 mM phenylmethanesulfonyl fluoride (PMSF) and protease inhibitor (MCE, NJ, USA). BCA protein assay kit (Generay Biotechnology, Shanghai, China) was used to quantified protein concentrations. Equal amount of protein was applied for SDS-PAGE electrophoresis and then transferred to PVDF membranes (Millipore, MA, USA). Membranes were blocked with 5% skim milk at room temperature for 1 h, then incubated with primary antibodies C3 (1:1000, Abcam), iNOS (1:1000, Abcam), S100A10 (1:1000, Abcam), Arg1 (1:1000, Cell Signaling Technology), suppressor of cytokine signaling 1 (SOCS1) (1:1000, Abcam) and β-actin (1:5000, Cell Signaling Technology) overnight at 4 °C. HRP-conjugated secondary antibodies were used for further incubation with the membranes for 120 min, and signals on blots were developed by the ECL reagents (Millipore, MA, USA) and were analyzed by ImageJ software.

### Real-time quantitative PCR analysis

Total RNA was extracted from cultured cells and brain tissues using TRIzol reagent according to the manufacturer’s protocol (Sigma-Aldrich, USA). The concentration of total RNA in each sample was measured using a NanoDrop 2000 Spectrophotometer (Thermo Fisher Scientific, MA, USA). For mRNA detection, equal amount of RNA was reverse transcribed into cDNA using RevertAid First Strand cDNA Synthesis Kit (Thermo Fisher Scientific, USA). Real-time quantitative PCR was performed with UltraSYBR Mixture (CWBio, Beijing, China) using the Mx3000P Real-Time PCR System (Agilent Technologies, USA). mRNA expression levels were normalized to the endogenous control β-actin. Relative mRNA expressions were quantified by 2^-ΔΔCt^ method. The primer pairs were listed in Table 1**.**

**Table 1:** Sequences of the primers used for detecting genes by qRT-PCR

Genes	Primer 5′	Primer 3′
C3	CACAGCGGCACATTTCAT	GGGGTCGGTCAAGGTCTA
iNOS	TGGTGGTGACAAGCACATTT	GTCATGAGCAAAGGCACAGA
CXCL10	TGCAAGTCTATCCTGTCCGC	ACGGAGCTCTTTTTGACCTTC
IL-1β	AATGCCTCGTGCTGTCTGACC	AGGGTGGGTGTGCCGTCTTT
TNF-α	AGAACTCCAGGCGGTGTCTGT	CTGCTCCTCTGCTTGGTGGTT
IL-12	TCACTTCGGCCAGGGTCATA	CCATGTCGTCCGTGGTCTTC
IL-23	GTGAGAAGCCTGGAGCATCA	AGGCAGTTACAGAGCTTCCG
S100A10	GAAAGGGAGTTCCCTGGGTT	CCCACTTTTCCATCTCGGCA
Arg1	TTGATGTTGATGGACTGGAC	TCTCTGGCTTATGATTACCTTC
MRC1	TCGCTGTTCAACTCTTGGGT	GCAGGCTCTATTCGAGTCAGG
IL-1ra	TGGAAATCTGCAGGGGACCT	CAGCAATGAGCTGGTTGTTCC
IL-10	CCTCTGGATACAGCTGCGAC	GTAGATGCCGGGTGGTTCAA
YM1	GGAAACTGGGTGCTACGAGA	GACCAGTTTGTACGCAGAGC
FIZZ1	GGCACGAGGGGACACTGA	GTGCATGAGAGGGTCTTCCT
β-actin	GGAGATTACTGCCCTGGCTCCTA	GACTCATCGTACTCCTGCTTGCTG

### Neurological deficit evaluation

The modified neurologic severity score (mNSS) was used to evaluate the neurobehavioral outcome at 7 days after MCAO as described in previous study [32]. There are four tests in the scoring systems, including motor, sensory, balance, and reflex tests. Scores from all the tests were summed together, where 0 represents no deficit and 14 represents maximal deficit.

### Morris water maze testing

Spatial learning and memory were evaluated using the Morris water maze (MWM) tests, which began on the 15th day after surgery [33]. The rats were placed in a circular pool 180 cm in diameter and 30 cm deep which was filled with 25 °C water to make the 9-cm platform 1.5 cm below the water surface. On the first 5 days, the rats were trained to find the platform in four trails per day. If rats failed to reach the platform within 90 s, they would be manually guided to the platform and remained there for 10 s before being placed back to cage. The latency and swimming path of reaching the platform were recorded for each rat and their declines over days of training reflected learning and memory. At last, the platform was removed to perform probe trial, and each rat was allowed to search the platform for 60 s. The escape latency to find the platform, the swim path length, the time spent in the target quadrant, and platform crossovers were tracked and analyzed by the ANY-maze video tracking software (Stoelting, USA).

### Statistical analysis

Statistical analyses were performed with GraphPad Prism software (version 6.0 c, GraphPad Software, Inc., La Jolla, CA) and SPSS (version 22, SPSS Inc., IBM, Armonk, NY). Parameters were expressed as mean ± SD. Behavioral test results were analyzed with repeated-measures analysis of variance (ANOVA) followed by Tukey post hoc test for each time point. Other results were analyzed with independent sample *t* test (for two groups) and one-way ANOVA followed by Tukey HSD post hoc test (for multiple groups). *P* < 0.05 was considered as statistically significant.

## Results

### rTMS effects on astrocytic survival and proliferation

**Fig. 2:**
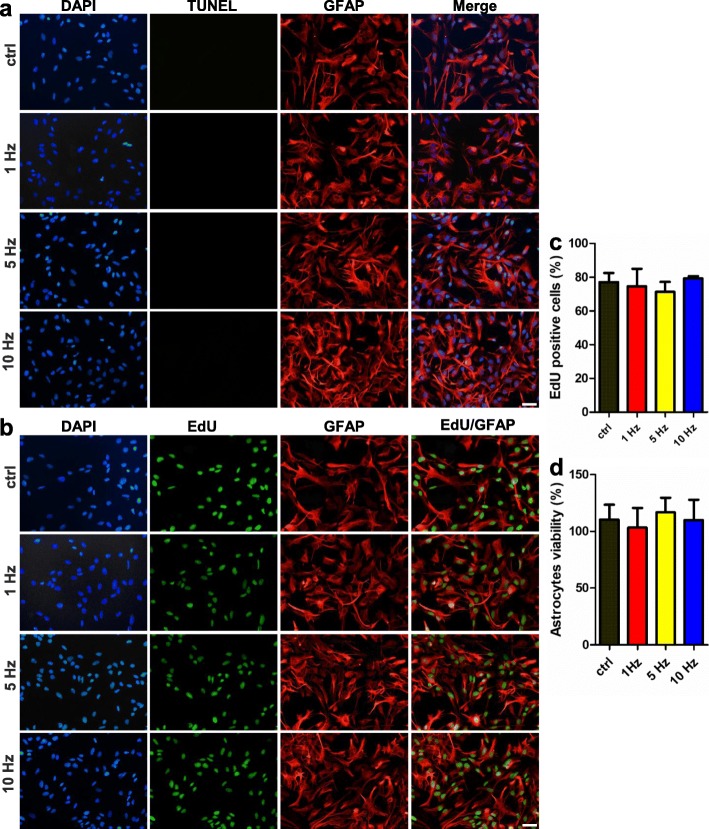
Cell survival and proliferation were not affected by rTMS. **a** Detection of apoptosis with terminal dUTP nick end-labeling (TUNEL) assay after receiving rTMS stimulation. No apoptotic signals were detected after rTMS stimulation with different frequencies. *n* = 5 per group. **b** Representative images of EdU staining post rTMS stimulation. **c** Quantification of the proportion of BrdU+/GFAP+ cells. *n* = 5 per group. **d** Detection of cell viability with CCK-8 experiment kit after receiving rTMS stimulation. rTMS stimulation with different frequencies did not affect astrocytes viability. CCK-8 experiment was repeated for three independent times and *n* = 5 per group. Scale bar: 50 μm. Data are represented as mean ± SD

rTMS is a noninvasive method to stimulate the brain. Three different frequencies of rTMS were first applied to primary astrocytes (Fig. 1a), and the influences on cell viability and proliferation were evaluated. As shown in Fig. 2a, no TUNEL-positive cells were detected after stimulation, indicating that no astrocytic apoptosis was triggered. The percentage of EdU-positive astrocytes were the same among the sham-stimulated group, 1 Hz group, 5 Hz group, and 10 Hz group, revealing that cell proliferation was not disturbed by the stimulations (Fig. 2b, c). The CCK-8 experiments showed similar cell viability after stimulation when compared to sham stimulated cells (Fig. 2d). Above data proved that rTMS protocols adopted in the present study had no harmful effects to primary astrocytes.

### rTMS counteracted OGD/R injury induced astrocytic alteration

**Fig. 3:**
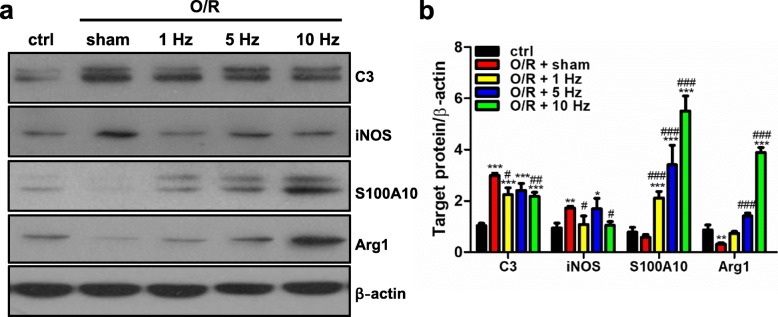
rTMS affected astrocyte phenotype following OGD/R injury. The primary astrocytes were exposed to OGD injury and received rTMS stimulation with different frequencies. **a** Western blot was used to detect the expression of neurotoxic markers (C3 and iNOS) and neuroprotective markers (S100A10 and Arg1). Images were representative of three independent experiments. **b** After OGD/R injury, C3 and iNOS were significantly increased, and Arg1 was significantly reduced. All of the rTMS frequencies could inhibit the expression of C3 and iNOS, and promote the expression of S100A10 and Arg1. The 10 Hz TMS induced the lowest expression of iNOS (1.04-fold) and the highest expression of S100A10 (5.51-fold) and Arg1(3.88-fold) among the OGD/R groups. *n* = 3 per group. Data are represented as mean ± SD. ** *P* < 0.01, *** *P* < 0.001 vs. ctrl astrocytes; ^#^*P* < 0.05, ^##^*P* < 0.01, ^###^*P* < 0.001 vs. O/R + sham stimulated astrocytes. O/R, oxygen and glucose deprivation/reoxygenation

The rTMS was applied to astrocytes following OGD/R injury, and the expression of polarization associated markers was detected. As shown in Fig. 3a, b, OGD/R significantly increased expression of neurotoxic markers (*P* < 0.001 for C3 and *P* = 0.002 for iNOS), and decreased expression of neuroprotective markers (*P* = 0.197 for S100A10 and *P* = 0.009 for Arg1). When rTMS applied, C3 and iNOS were reduced, S100A10 and Arg1 were significantly increased when compared with OGD/R + sham stimulation group. The 10 Hz rTMS had the most profound effects. The 10 Hz rTMS reduced C3 from 2.99 ± 0.05-fold to 2.18 ± 0.08-fold (*P* = 0.001), and reduced iNOS from 1.72 ± 0.04-fold to 1.05 ± 0.08-fold (*P* = 0.002), but increased S100A10 from 0.58 ± 0.06-fold to 5.51 ± 0.34-fold (*P* = 0.0001), and increased Arg1 from 0.32 ± 0.03-fold to 3.88 ± 0.11-fold (*P* < 0.0001).

**Fig. 4:**
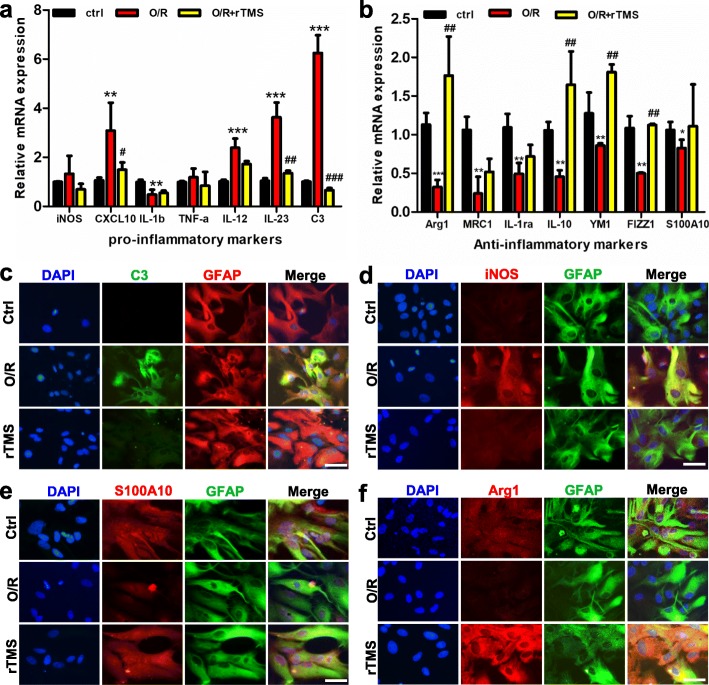
rTMS stimulation at 10 Hz inhibited neurotoxic and promoted neuroprotective polarization of primary astrocytes exposed to OGD/R injury. **a**, **b** mRNA expression of neurotoxic markers and neuroprotective markers in different groups (control, O/R, and O/R + rTMS) detected by qRT-PCR at 48 h post-reoxygenation. *n* = 3 per group. **c**, **d** Immunofluorescent staining of GFAP and neurotoxic markers (C3 and iNOS), anti-DAPI (blue) after indicated treatments. **e**, **f** Immunofluorescent staining of GFAP and neuroprotective markers (S100A10 and Arg1), anti-DAPI (blue) after indicated treatments. Scale bar = 50 μm. Images were representative of three independent experiments. *n* = 3 per group. Data are represented as mean ± SD; * *P* < 0.05, ** *P* < 0.01, *** *P* < 0.001 vs. control astrocytes. ^#^*P* < 0.05, ^##^*P* < 0.01, ^###^*P* < 0.001 vs O/R astrocytes. *O*/*R* oxygen and glucose deprivation/reoxygenation

The effects of the 10 Hz rTMS on astrocytic polarization were further verified by detecting the mRNA expression level and immunofluorescence of phenotypic markers. As shown in Fig. 4a, the neurotoxic markers, including C3, iNOS, CXCL-10, IL-1b, TNF-α, IL-12, and IL-23, significantly increased after OGD/R injury, but these reversed to the baseline after treating with 10 Hz rTMS. Furthermore, there was an obvious decrease in alternatively activated markers, including S100A10, Arg1, mannose receptor c-type 1 (MRC1), IL-1ra, IL-10, YM1, and found in inflammatory zone 1 (FIZZ1), after OGD/R, and this was rescued by the rTMS treatment (Fig. 4b). In the immunofluorescent experiment, OGD/R injury significantly increased C3 and iNOS expression, and this was inhibited by rTMS treatment (Fig. 4c, d). Meanwhile, S100A10 and Arg1 (the neuroprotective indicators) significantly decreased after OGD/R exposure, and this was rescued by rTMS treatment (Fig. 4e, f). These results were consistent with the changes in protein expression levels (Fig. 3). The above data indicated that 10 Hz of rTMS had the most potential to inhibit neurotoxic polarization, and promote neuroprotective polarization of astrocytes after OGD/R injury among the three frequencies.

### rTMS alleviated neurotoxic effects of astrocytes following OGD/R injury

**Fig. 5:**
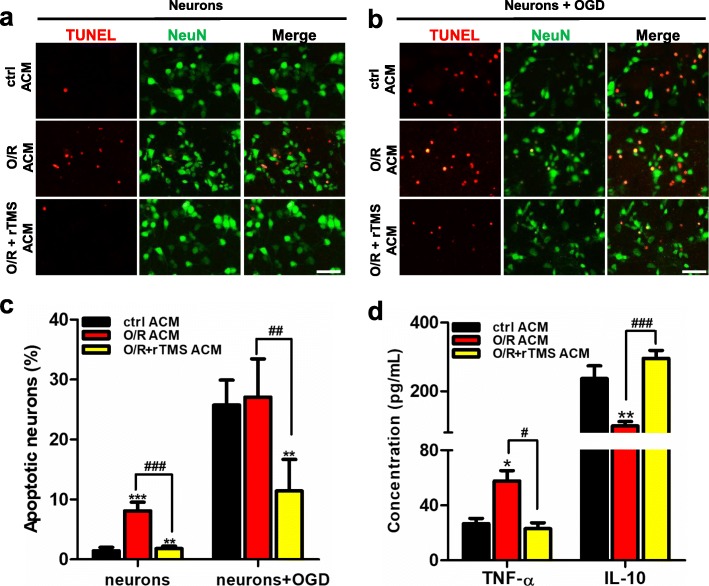
rTMS inhibited astrocytic secretion of pro-inflammatory mediators to alleviated neuronal death. Normal neurons and post-OGD neurons were co-cultured with the astrocytes culture medium (ACM) collected from indicated groups for 48 h. TUNEL staining was used to detect the apoptosis of neurons (**a**) and neurons exposed to OGD/R injury (**b**), with scale bar of 20 μm. Images were representative of three independent experiments. **c** The statistical results of TUNEL showed that O/R + rTMS ACM group induced less apoptosis than the O/R ACM group in normal neurons (*P* < 0.001) and post-OGD neurons (*P* < 0.01). **d ** Concentration of TNF-α and IL-10 in the ctrl ACM, O/R ACM, and O/R + rTMS ACM were detected by ELISA kits. O/R significantly induced TNF-α (*P* < 0.05) but reduced IL-10 secretion (*P* < 0.01), while TMS inhibited TNF-α (*P* < 0.05) but promoted IL-10 secretion (*P* < 0.001). *n* = 6 per group. Data are represented as mean ± SD; *P < 0.05, **P < 0.01, ***P < 0.001 vs. ctrl ACM group. ^#^*P* < 0.05, ^##^*P* < 0.01, ^###^*P* < 0.001 vs. O/R ACM group. *O*/*R* oxygen and glucose deprivation/reoxygenation

To elucidate the effects of rTMS-regulated astrocytes on neuronal survival under hypoxic/ischemic conditions, astrocytic culture medium (ACM) was collected from control astrocytes, OGD/R + sham stimulation astrocytes, and OGD/R + rTMS astrocytes. Then, these ACM were mixed with neuronal culture medium (1:1) to culture non-OGD or post-OGD neurons. Neuronal survival was ascertained 48 h later. The addition of OGD/R + sham stimulation ACM reduced survival of non-OGD and post-OGD neurons when compared with control astrocytes ACM (Fig. 5a–c). However, the OGD/R + rTMS ACM exerted neuroprotective effects, leading to the least neuronal apoptosis post oxygen-glucose deprivation and reoxygenation (O/R) injury.

In order to explain how ACM affected neuronal survival, the concentration of pro- and anti-inflammatory mediators in the ACM were detected. IL-10 is a typical anti-inflammatory mediator, and TNF-α is a representative pro-inflammatory mediator. The ELISA results revealed that astrocytic secretion of TNF-α increased from 26.52 pg/mL to 57.73 рg/mL (*P* = 0.02), while the secretion of IL-10 decreased from 236.9 рg/mL to 99.03 рg/mL (*P* = 0.007) after OGD/R injury. However, rTMS reduced the concentration of TNF-α to 22.95 рg/mL, but increased IL-10 secretion to 555.1 рg/mL (*P* = 0.004 when compared with control group, Fig. 5d). These results demonstrated that rTMS reversed neurotoxic effect of OGD/R astrocytes to neuroprotective effect.

### Astrocytes promoted synaptic formation after receiving rTMS stimulation

**Fig. 6:**
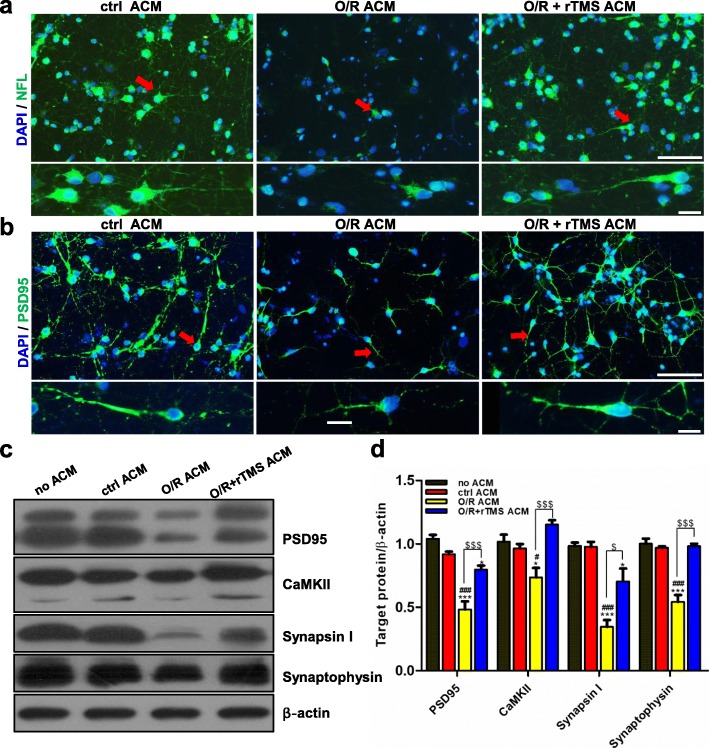
Astrocytes regulated by rTMS increased axonal density and promoted neural plasticity following OGD/R injury. The anoxic neurons were co-cultured with ACM collected from different groups for 7 days. Axonal density was represented by neurofilament (NFL) staining (**a**) and synaptic plasticity was reflected by PSD95 staining (**b**). Scale bar: 50 μm. Images were representative of three independent experiments. **c**, **d** Western blots and quantification of synaptic markers which included PSD95, CaMKII, Synapsin I, and Synaptophysin. *n* = 3 per group. Data are represented as mean ± SD; *P < 0.05, **P < 0.01, ***P < 0.001 vs. no ACM group. ^#^*P* < 0.05, ^##^*P* < 0.01, ^###^*P* < 0.001 vs. ctrl ACM group.^$^*P* < 0.05, ^$$^*P* < 0.01, ^$$$^*P* < 0.001 vs. O/R ACM group. *O*/*R* oxygen and glucose deprivation / reoxygenation

Previous studies have indicated that rTMS-regulated astrocytes could promote synaptic formation and nerve growth [34]. Thus, it was tested whether rTMS-modulated OGD/R astrocytes still has these functions. OGD/R neurons were co-cultured by mixture of ACM and normal neuronal culture media for 7 days. Immunofluorescence experiments showed that OGD/R ACM reduced the axonal density (neurofilament staining, NFL) and the length of synapses (PSD95 staining) when compared with control ACM cultured neurons. However, the OGD/R + rTMS ACM alleviated the loss of axons and synapses **(**Fig. 6a, b). The expression of synaptic proteins, including PSD95, CaMK II, Synapsin I, and Synaptophysin in neurons, was also assessed. The rTMS-stimulated ACM was associated with substantial high levels of all these proteins (Fig. 6c, d, *P* < 0.05). So, the long-term treatment with rTMS-stimulated ACM promoted the axonal density and synaptic plasticity.

### rTMS effects on astrocytes were counteracted by LPS treatment

**Fig. 7:**
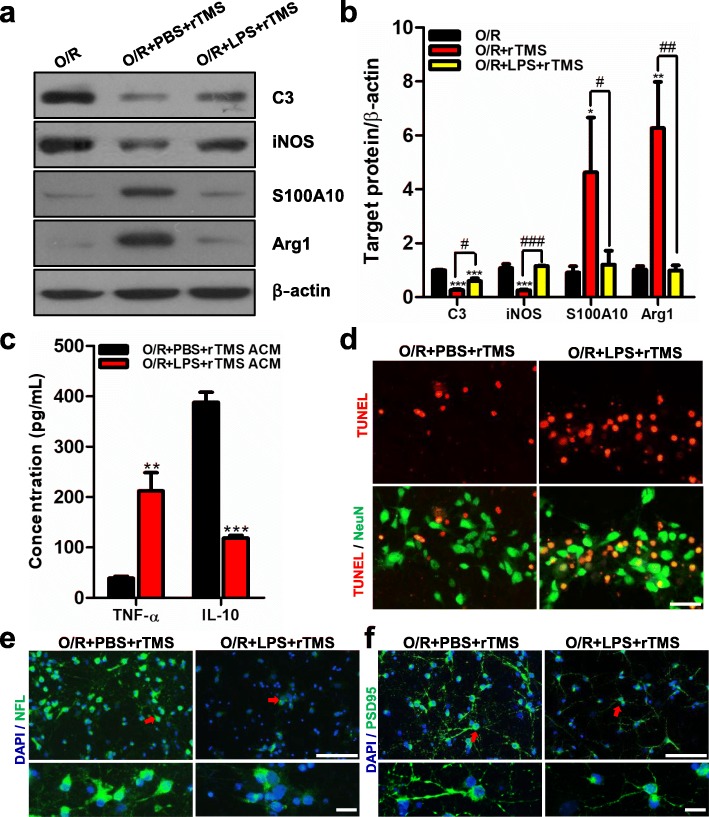
LPS antagonized rTMS effects on astrocytes and weakened rTMS-related neuroprotective effects. After exposing to OGD, primary astrocytes received rTMS stimulation and being cultured with LPS (100 ng/mL) for 8 h. Then, cells were cultured with normal medium. Proteins and ACM were obtained at 48 h-post OGD. **a** Expression of neurotoxic markers (C3 and iNOS) and neuroprotective markers (S100A10 and Arg1) were detected by Western blot. Images were representative of three independent experiments. **b** Quantification of indicated markers by ImageJ. *n* = 3 per group. Data are represented as mean ± SD; ***P < 0.001 vs. O/R group. ^#^*P* < 0.05, ^#^*P* < 0.01, ^###^*P* < 0.001 vs. O/R + rTMS group. **c** TNF-α and IL-10 concentration in the indicated ACM were detected by ELISA experiment. *n* = 5 per group. Data are represented as mean ± SD; **P < 0.01, ***P < 0.001 vs. PBS + O/R + rTMS group. **d** TUNEL (red) and NeuN (green) staining of normal neurons co-cultured with indicated ACM for 48 h. *n* = 3 per group. Neurofilament (**e**) and PSD95 (**f**) staining of normal neurons co-cultured with ACM for 7 days. The red arrows indicated the areas that magnified on the bottom panels. Scale bar: 50 μm. *n* = 3 per group. *O*/*R* oxygen and glucose deprivation/reoxygenation

LPS has been reported to induce the pro-inflammatory transition of astrocytes [35]. Hence, astrocytes were stimulated with LPS to explore whether rTMS effects on astrocytes would be antagonized. As shown in Fig. 7a, b, the PBS treatment did not affect the rTMS effects on OGD/R astrocytes. C3 and iNOS were significantly reduced, and S100A10 and Arg1 were markedly increased by rTMS in the PBS-treated group. However, when LPS and rTMS were synchronously applied to OGD/R astrocytes, no significant changes were detected between OGD/R and OGD/R + LPS + rTMS group, indicating that the rTMS effects of promoting anti-inflammatory transformation of astrocytes was counteracted by LPS treatment. The TNF-α concentration in ACM increased from 39.08 to 212.1 pg/mL (*P* = 0.002), while IL-10 concentration reduced from 387.7 to 118.3 pg/mL (*P* < 0.0001), when comparing LPS-treated group with the PBS-treated group (Fig. 7c). The ACM collected from the LPS-stimulated group induced more TUNEL positive neurons than in the PBS group (Fig. 7d). The long-term co-culture with ACM lost the ability to promote axon growth and synaptic formation when using ACM collected from LPS group. The axonal density (NFL staining) was lower (Fig. 7e) and the length of synapses (PSD95 staining) was shorter and thinner (Fig. 7f) in the LPS group than in the PBS group. These above data indicated that rTMS induced astrocytic transition and the associated neuroprotective effects were weaken or almost abolished by LPS treatment. This proved that rTMS was specific for promoting anti-inflammatory transformation of astrocytes.

### Neurological deficits following ischemic stroke were attenuated by rTMS stimulated ACM

**Fig. 8:**
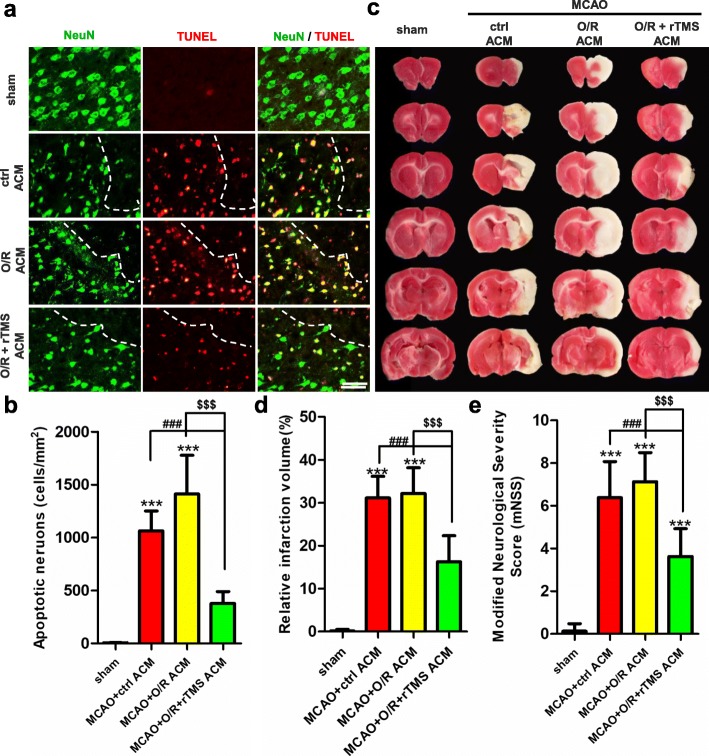
The injection of rTMS stimulated ACM reduced neuronal apoptosis, infarct volumes, and promoted functional recovery in MCAO rats. Immediately after reperfusion and at 3 days post reperfusion, the MCAO rats received retroorbital intravenously injection of indicated ACM. At 7 days post reperfusion, apoptotic neurons were detected by NeuN/TUNEL (**a**), with scale bar of 50 μm. *n* = 3 animals per group. **b** Positive apoptotic neurons were calculated using ImageJ in at least five random fields. **c**Representative image of TTC staining in the indicated groups. *n* = 8 animals per group. **d** Quantitative analysis of infarct volume, *n* = 8. **e**The neurological deficit was evaluated by modified Neurological Severity Score (mNSS). *n* = 8 animals per group. Data are represented as mean ± SD; *P < 0.05, **P < 0.01, ***P < 0.001 vs. sham rats. ^###^*P* < 0.001 vs. MCAO + sham ACM group. ^$$$^*P* < 0.001 vs. MCAO + O/R ACM group. *O*/*R* oxygen and glucose deprivation/reoxygenation

Next, it was determined whether the neuroprotective effects of rTMS-regulated ACM could be observed in vivo. MCAO rats were randomly subjected into ctrl ACM, OGD/R ACM, and OGD/R + rTMS ACM group, receiving retroorbital intravenous injection of indicated ACM at the time of reperfusion, and at 3 days after reperfusion. TUNEL/NeuN staining results manifested that the OGD/R ACM aggravated neuronal apoptosis, while the OGD/R + rTMS ACM treatment mitigated neuronal apoptosis in the peri-infarct region 7 days after reperfusion (Fig. 8a, b, *P* < 0.001). Consistent with the TUNEL staining results, the OGD/R ACM treatment lightly increased infarct volume, while the OGD/R + rTMS ACM administration significantly reduced infract volume from 31.14 ± 1.77% to 16.25 ± 2.15%, when compared to ctrl-ACM-treated MCAO rats (Fig. 8c, d, *P* = 0.0001). Besides, the mNSS score for the OGD/R ACM-treated group was the highest, while the OGD/R + rTMS ACM-treated group was the lowest among the MCAO rats (Fig. 8e), indicating that OGD/R ACM exacerbated neurological deficits while rTMS stimulated ACM attenuated these neurological deficits.

**Fig. 9:**
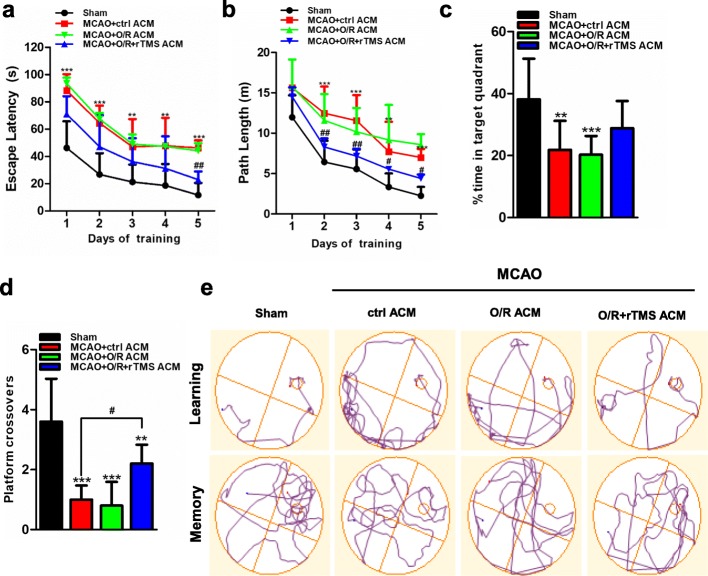
Administration of ACM collected from rTMS-regulated astrocytes alleviated ischemia/reperfusion (I/R)-induced cognitive deficits. At 14 days post reperfusion, rats in indicated groups were applied for Morris water maze (MWM) tests. Latencies (**a**) and swimming path length (**b**) to find the platform in the learning stage. **c** Percentage of time spent in the platform quadrant in the probe trial. **d** The frequency of swimming across the platform location in the probe trial. **e** Representative track plots of rats in hidden platform test (top plots, “learning”) and probe trial stage (bottom plots, “memory”). Data are shown as mean ± SD; *n* = 10 per group; ***P < 0.001 vs. sham rats; ^#^*P* < 0.05, ^##^*P* < 0.01, ^###^*P* < 0.05 vs. MCAO + ctrl ACM group. *O*/*R* oxygen and glucose deprivation/reoxygenation

The Morris water maze (MWM) test was also used to assess the long-term neurological functions. In the spatial learning stage, all rats from different groups benefited from the 5-day training, and exhibited gradually decreased latency and swim path to the platform (Fig. 9a, b  and e). The performance by the MCAO rats was worse than that of sham-operated rats. rTMS stimulated ACM improved spatial memory by shortening the escape latency and swim path to the platform, and significant differences were presented on the fifth day (*P* < 0.01 for escape latency, *P* < 0.05 for path length). In the spatial probe trial, the frequency of swimming across the platform and percentage of time spent in the platform quadrant were the representations of cognitive performance (Fig. 9c, d  and e). The OGD/R + rTMS ACM alleviated the MCAO-induced reduction on the platform crossovers (2.20 ± 0.20 vs. 1.00 ± 0.15, *P* < 0.05). The percentage of time spent in the target quadrant was higher in the OGD/R + rTMS ACM group than in the control ACM group (28.78 ± 2.27 vs. 21.87 ± 2.95, *P* = 0.07), while OGD/R ACM injection lightly exacerbated MACO-induced cognitive deficits. These results proved that rTMS treatment could alleviate neuronal apoptosis and promote neurological functional recovery through inhibiting neurotoxic polarization of astrocytes.

### rTMS lightened A1 astrocytic motivation in ischemic rats

**Fig. 10:**
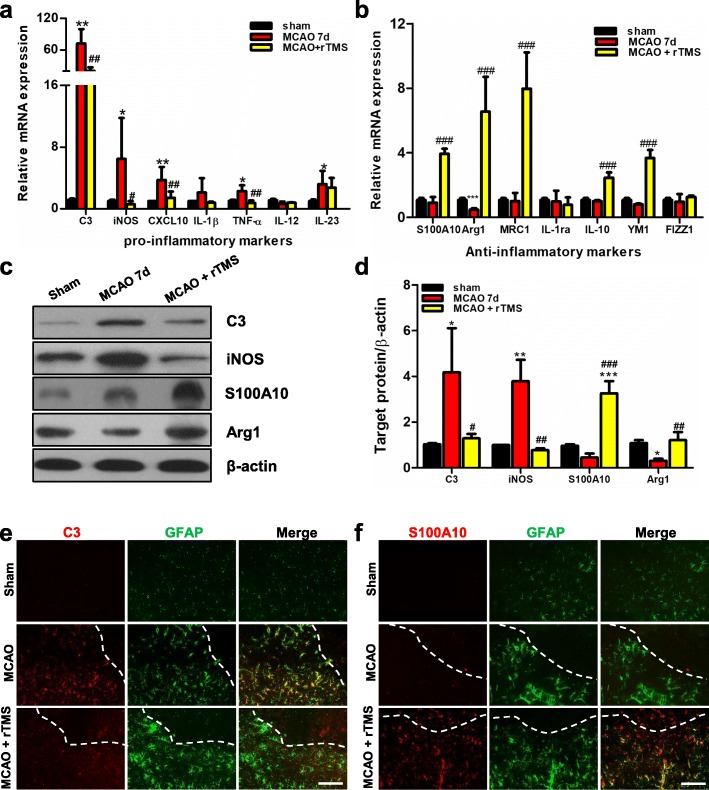
rTMS stimulation at 10 Hz inhibited neurotoxic polarization but promoted neuroprotective transformation of astrocytes in the ischemic penumbra. **a**, **b** mRNA expression levels of neurotoxic markers and neuroprotective markers in different groups (sham operated, MCAO, MCAO + rTMS) detected by qRT-PCR at 7 days post ischemic reperfusion. *n* = 5 animals per group. **c**, **d** Representative immunoblots and quantification of neurotoxic markers (C3 and iNOS) and neuroprotective markers (S100A10 and Arg1) in lysates of brain tissue obtained from ischemic penumbra with different treatments. β-actin was used as a loading control. *n* = 5 animals per group. **e**, **f** Representative double-staining immunofluorescence of C3^+^/GFAP^+^ and S100A10^+^/GFAP^+^ astrocytes on brain sections obtained from Sham, MCAO or MCAO + rTMS. Scale bar = 50 μm, *n* = 5 per group. Data are represented as mean ± SD; *P < 0.05, **P < 0.01, ***P < 0.001 vs. sham-operated rats. ^#^*P* < 0.05, ^##^*P* < 0.01, ^###^*P* < 0.001 vs. MCAO rats

After verifying the rTMS effects to reverse neurotoxic astrocytes into neuroprotective astrocytes in vitro, it was determined whether rTMS had similar effects in vivo. The 10 Hz rTMS was delivered at 24 h post ischemic-reperfusion for 10 min every day, and this was sustained for seven consecutive days. Proteins and RNAs from sham, MCAO, and MCAO + rTMS groups were applied for Western blot and quantitative real-time reverse transcription PCR (qRT-PCR) analysis. In MCAO rats, mRNA level of neurotoxic markers, except for IL-12, all significantly increased, and most of the anti-inflammatory markers remained around the baseline level at 7 days after reperfusion (Fig. 10a, b). In the MCAO + rTMS group, the neurotoxic markers were around the baseline level, while the neuroprotective markers were dramatically higher than those in the sham group. The protein expression was consistent with that in the mRNA results (Fig. 10c, d). C3 increased to 4.18-fold (*P* = 0.047) and iNOS increased to 3.89-fold (*P* = 0.007), while Arg1 decreased to 0.308-fold (*P* < 0.001) in MCAO rats. When rTMS was applied, MCAO increased C3 and iNOS were dramatically decreased to the baseline, and MCAO inhibited S100A10 and Arg1 were remarkably rescued. The immunofluorescence staining showed that most of GFAP^+^ cells in the penumbral region were co-stained with C3 (Fig. 10e), but very few co-stained with S100A10 (Fig. 10f) in MCAO group at day 7 post-ischemic injury. However, in MCAO + rTMS group, most GFAP^+^ cells were co-stained with S100A10 (Fig. 10f), instead of C3 (Fig. 10e). So, the in vivo data supported the present finding that rTMS could inhibit the neurotoxic polarization of astrocytes, maintaining astrocytes in neuroprotective phenotype.

## Discussion

The present study demonstrated that 10 Hz rTMS could reduce infarct volumes and improve functional recovery through reversing neurotoxic astrocytes to neuroprotective astrocytes following OGD/R and ischemia/reperfusion (I/R) injury.

Different frequencies of rTMS can produce different modulatory effects [8]. The present study assessed three different frequencies (1 Hz, 5 Hz, and 10 Hz) of rTMS protocols. The results revealed that cell survival and proliferation were not affected by rTMS stimulation, which was consistent with previous reports [12]. However, there were frequency-specific effects of rTMS on phenotypic status of astrocytes following OGD/R injury. Compared with other frequencies, the 10 Hz rTMS may be most efficacious in transforming neurotoxic astrocytes into neuroprotective phenotypes. The present conclusion appeared to be inconsistent with a meta-analysis, which revealed that low-frequency rTMS over the unaffected hemisphere was more beneficial than high-frequency rTMS over the affected hemisphere [5]. In fact, the OGD/R model only modified the status in the affected hemisphere. The present finding explained why (from the perspective of astrocytes) clinical high-frequency rTMS is often chosen over low frequency when stimulating the lesion side. Besides, functions of astrocytes in the unaffected hemisphere were changed following ischemic stroke [36]. Whether the regulation of astrocytes is also involved in the more beneficial low-frequency rTMS stimulation on the unaffected hemisphere requires further study. To date, the conclusions about the frequency-specific effects on promoting functional recovery for ischemic stroke patients remain controversial. Despite the effectiveness of low-frequency reported in the meta-analysis, the recently conducted NICHE Trial revealed no differences between the 1 Hz and sham rTMS trial arms [37]. As discussed in the NICHE Trial, the lack of difference does not imply that rTMS has no role in improving recovery. A variety of technical variables, including the stimulation locations, the type of coils used, the duration and number of treatments, and the functional assessment tools, might lead to differences observed in different research results.

Astrocytes are important regulatory cells with multi functions in the CNS, and are regarded as highly plastic cells that exhibit various structural and functional changes in response to the insults that alter their environment [38, 39]. Recently identified A1 and A2 phenotypes of astrocytes are similar to the previously defined classically activated and alternatively activated astrocytes [23–25]. A1, the classically activated astrocytes, can produce pro-inflammatory mediators which may damage neurons and oligodendrocytes. While A2, the alternatively activated astrocytes, can secrete anti-inflammatory mediators which may promote neuronal survival and tissue repair. In our studies, we classified astrocytes into neurotoxic phenotype and neuroprotective phenotype with markers used by A1/A2 and classically/alternatively activation. Although some studies have indicated that ischemia induced A2 astrocytic activation [40], other researchers reported that a large number of neurotoxic genes were also induced at the same time in astrocytes [26]. Oxygen species, pro-inflammatory cytokines, and interleukins were secreted by astrocytes to amplify ischemic injury [41–43]. The present results draw parallels with the most recently published article that detected classically activated astrocytes in mice with MCAO, exerting harmful effects to anoxic neurons after brain ischemia [25]. Discovering methods to prevent neurotoxic phenotype polarization while maintaining neuroprotective functions of astrocytes holds great potential for facilitating repair and recovery following ischemic injury.

In the present study, rTMS is a good candidate to achieve this aim, particularly when delivered at 10 Hz frequency. Despite the fact that most of rTMS studies were neuron-centric, research conducted over the past two decades has made it increasing clear that astrocytes are likely cellular effectors of rTMS [13]. The 1 Hz stimulation lead to statistically significant rise in intracellular calcium of the cultured astrocytes [12]. Astrocyte hypertrophy was reduced in response to rTMS at 24 h post-scratch injury. In the EAE model, rTMS reduced the neuroinflammation, which was mediated by astrocytes [44]. However, there is a lack of data pertaining to the astrocytes when rTMS treat ischemic stroke. The present study was the first to reveal that rTMS could reverse neurotoxic astrocytes to neuroprotective astrocytes in cerebral ischemic stroke model. The OGD/R or I/R-induced A1 marker C3 and classically activated marker iNOS were significantly inhibited after receiving 10 Hz rTMS. The cell culture media collected from rTMS stimulated astrocytes alleviated OGD/R-induced neuronal apoptosis. Previous studies have proved that rTMS has a direct effect on neurons in reducing neuronal death, while in our study, it was found that rTMS could also work on astrocytes to indirectly reduce neuronal death. Besides, astrocytes regulated by rTMS promoted synapse formation. MCAO rats that received rTMS-stimulated ACM had smaller infarct volumes and better cognitive function recovery. Supporting these findings, rTMS stimulation has been reported to regulate post-synaptic morphology and function by mediating Ephrin A3, which is highly expressed by astrocytes [45, 46]. The therapeutic effect of rTMS in attenuating depression has been largely attributed to the astrocytic involvement in mediating synaptic structure and efficacy [13, 47, 48]. Therefore, the mechanisms by which rTMS promoted the cerebral ischemic brain recovery might be in relation to the modulation of astrocytic phenotypes. In addition, the microglia are another type of glia cell, which has been also reported to induce neuroinflammation during stroke [49]. The effect of rTMS on microglia remains largely unexplored. However, low-intensity rTMS has been reported to affect macrophage/microglia infiltration in a rat model of cerebral ischemia [50]. Further studies are needed to determine whether and how the 10-Hz rTMS protocol reported in the present study affects microglia.

Several limitations are inherent in the present study. First, we only demonstrated a secondary neuroprotective effect of rTMS by regulating astrocytes in vitro, but did not test the neuroprotective effect of rTMS in MCAO rats that directly received rTMS. Since the rTMS itself has anti-apoptosis and pro-synaptic functions on neurons, and the secondary neuroprotective effect induced by rTMS-regulated astrocytes could not be distinguished from the rTMS direct effect on neurons [51, 52]. Hence, exogenous ACM was injected to the lesion site. In this way, it could be specifically proven that these neuroprotective effects were triggered by rTMS-regulated astrocytes. Second, the underlying mechanism about how rTMS inhibit neurotoxic astrocytes in cerebral ischemia remains unknown. NF-kB and STAT3 pathways were involved in the A1 polarization of astrocytes, controlling the expression of numerous pro-inflammatory and neurotoxic mediators [53, 54]. LCN2 inhibited the phenotypic change of astrocytes toward anti-inflammatory activation [55]. Micro-RNA155 and its star-form partner miR-155* have been reported to promote A1 transformation [56]. Numerous signaling pathways have been reported to be involved in the neurotoxic polarization of astrocytes, but which molecular markers played the key role remained undetermined [23]. To explore the signaling pathway affected by rTMS to inhibit the neurotoxic astrocytes, we first need to find the key switch that promotes the neurotoxic polarization of astrocytes.

## Conclusions

The present study demonstrated that 10 Hz rTMS could inhibit neurotoxic transformation of astrocytes after focal cerebral ischemia. Astrocytes modulated by the 10 Hz rTMS gained anti-inflammatory and pro-synaptic functions. These results illustrated a novel mechanism for the effectiveness of rTMS in treating ischemic stroke.

## Data Availability

The datasets used and/or analyzed during the current study are available from the corresponding author on reasonable request.

## Abbreviations

ACM: Astrocytes-conditioned media
Arg1: Arginase 1
C3: Compliment C3
FIZZ1: Found in inflammatory zone 1
GFAP: Glial fibrillary acidic protein
I/R: Ischemia/reperfusion
IL-1β: Interleukin 1 beta
iNOS: Inducible nitric oxide synthase
MCAO: Middle cerebral artery occlusion
mNSS: Modified neurologic severity score
MRC1: Mannose receptor c-type 1
O/R: Oxygen-glucose deprivation and reoxygenation
OGD: Oxygen-glucose deprivation
OGD/R: Oxygen-glucose deprivation and reoxygenation
qRT-PCR: Quantitative real-time reverse transcription PCR
S100A10: S100 calcium binding protein A10
SOCS1: Suppressor of cytokine signaling 1
TNF-α: Tumor necrosis factor alpha
TUNEL: Transferase-mediated deoxyuridine triphosphate-biotin nick end labeling
